# Metformin enhances the radiosensitizing effect of cisplatin in non-small cell lung cancer cell lines with different cisplatin sensitivities

**DOI:** 10.1038/s41598-018-38004-5

**Published:** 2019-02-04

**Authors:** Muhammad Assad Riaz, Ali Sak, Yasin Bahadir Erol, Michael Groneberg, Jürgen Thomale, Martin Stuschke

**Affiliations:** 1 Department of Radiotherapy, University Hospital Essen, Essen, Germany; 2 Institute of Cell Biology, University Hospital Essen, Essen, Germany

## Abstract

Cisplatin is an extensively used chemotherapeutic drug for lung cancer, but the development of resistance decreases its effectiveness in the treatments of non-small cell lung cancer (NSCLC). In this study, we examined the effects of metformin, a widely used antidiabetic drug, on cisplatin radiosensitization in NSCLC cell lines. Human NSCLC cell lines, A549 (cisplatin-resistant) and H460 (cisplatin-sensitive), were treated with metformin, cisplatin or a combination of both drugs before ionizing radiation. Cell proliferation, clonogenic assays, western blotting, cisplatin**-**DNA adduct formation and immunocytochemistry were used to characterize the treatments effects. Metformin increased the radiosensitivity of NSCLC cells. Metformin showed additive and over-additive effects in combination with cisplatin and the radiation response in the clonogenic assay in H460 and A549 cell lines (p = 0.018 for the interaction effect between cisplatin and metformin), respectively. At the molecular level, metformin led to a significant increase in cisplatin-DNA adduct formation compared with cisplatin alone (p < 0.01, ANOVA-F test). This was accompanied by a decreased expression of the excision repair cross-complementation 1 expression (ERCC1), a key enzyme in nucleotide excision repair pathway. Furthermore, compared with each treatment alone metformin in combination with cisplatin yielded the lowest level of radiation**-**induced Rad51 foci, an essential protein of homologous recombination repair. Ionizing radiation-induced γ-H2AX and 53BP1 foci persisted longer in both cell lines in the presence of metformin. Pharmacological inhibition of AMP-activated protein kinase (AMPK) demonstrated that metformin enhances the radiosensitizing effect of cisplatin through an AMPK-dependent pathway only in H460 but not in A549 cells. Our results suggest that metformin can enhance the effect of combined cisplatin and radiotherapy in NSCLC and can sensitize these cells to radiation that are not sensitized by cisplatin alone.

## Introduction

Cisplatin is a first**-**line chemotherapeutic agent that is often used in combination with third generation cytotoxic agents such as gemcitabine, taxanes or vinca alkaloid to treat a wide variety of tumors including NSCLC^1^. Cisplatin binds with DNA and forms cisplatin-DNA-adducts, which are largely responsible for much of the cellular cytotoxicity of this drug. Previous studies have demonstrated that the anti-tumor effect of cisplatin can be improved by multiple strategies in irradiated as well as in non- irradiated tumors^2,3^. A more recent study showed that suppressing the expression of key components of the nucleotide excision repair (NER) pathway, e.g. excision repair cross complement-1 (ERCC1) and x-ray repair cross complementing-1 (XRCC**-**1), aggravates the chemo**-** and radiosensitizing effects of cisplatin in head and neck cancer^4^. It is widely accepted that cisplatin-adducts formation inhibits DNA replication and transcription initiating a number of cellular responses that ultimately lead to cell death and apoptosis. Therefore, combining cisplatin with radiation therapy may represent a potential approach to improve the median survival of cancer patients. However, cisplatin efficacy in cancer treatment is limited due to drug resistance, which leads to treatment failure in many patients. Several factors are involved in the development of cisplatin resistance. Among them, the ability to repair cisplatin-DNA adducts appears to be of particular importance^5,6^. It is well established that most of the cisplatin-DNA adducts are mainly repaired by the NER pathway^7,8^. The over-expression of ERCC1, an essential endonuclease of this pathway, has been associated with cellular resistance to platinum**-**based chemotherapy in different cancers suggesting that platinum**-**based chemotherapy would be more effective in ERCC1-negative cancers^9^. Other studies have also clearly shown a positive association of higher ERCC1 expression with the DNA repair ability in cancer patients that might possibly be one of the explanations of resistance to platinum-based treatments^10–12^. Moreover, low levels of ERCC1 expression were associated with the improved response to platinum compounds in NSCLC, ovarian and breast cancer cells^13^. These data reveal a crucial role of the NER pathway and highlights the ERCC1 gene as an attractive molecular target to increase the cytotoxic effects of platinum compounds and overcome their resistance.

One area of great interest is to develop innovative drugs as well as novel therapeutic approaches to improve the sensitivity to platinum compounds and overcome their resistance in cancer patients. In this regard, multiple drugs were tested as cisplatin sensitizers over the past two decades^14–17^. However, currently there is no widely accepted application available that is effective in inhibiting the tumor progression in platinum-resistant disease. Metformin, a well-tolerated biguanide derivative, has been used for more than 50 years in clinical practice for the treatment of type 2 diabetes mellitus. Interestingly, numerous studies have confirmed the strong anti-cancer properties of metformin and suggested that it may improve the prognosis of patients with multiple cancers and prevent the tumor initiation^18–20^. Metformin inhibits the proliferation, cell survival and induces apoptosis in multiple cancer cells including lung cancer^21–23^. Metformin has also been previously shown to increase cisplatin cytotoxicity of H1975 and A549 cells mainly through inhibition of thymidine phosphorylase and ERCC1 proteins expression^24^. Moreover, results from a recent *in vitro* study using PC-9 and HCC-827 adenocarcinoma cells also suggested that metformin prevents and reverses resistance to gefitinib and cisplatin by decreasing the programmed death-ligand 1 expression^25^. Metformin was also shown to activate AMPK^26^, which was prevented in cells exposed to the AMPK inhibitor compound C. Further reports suggest that metformin can amplify chemotherapy-induced AMPK activation and enhances the cytotoxicity of chemotherapy in various cancer models^27,28^. It was previously documented that ionizing radiation activates the energy sensor AMP-activated kinase (AMPK) pathway in NSCLC^29,30^. AMPK induces p53 and cyclin-dependent kinase inhibitors p21cip1 and p27kip1, leading to cycle arrest^31,32^ through a DNA damage response (DDR)-mediated ataxia telengiectasia mutated (ATM) pathway and thus mediate radiosensitization^29^.

To assess the potential use in chemotherapy, anti-cancer effects of metformin have been tested in combination with several drugs as well as with radiotherapy. In fact, both *in vitro* and *in vivo* studies reveal that effectiveness of multiple chemotherapeutic drugs was markedly enhanced when co-administered with metformin with or without radiation^30,33–35^. A previous *in vitro* study performed on human lung cancer cell lines clearly reported that metformin in combination with cisplatin yielded stronger inhibitory effects on cell proliferation when compared with cisplatin treatment alone, mainly due to inhibiting the metabolic activity of these cells^36^. Furthermore, metformin has also been shown to greatly enhance the cytotoxic effects of paclitaxel, an antineoplastic drug, in human NSCLC cells through inhibiting the ERCC1 protein expression^37^. The critical role of this enzyme, as pointed out above, is to remove the cytotoxic cisplatin-adducts from cellular DNA through the NER pathway^13,38^, which makes this structure specific endonuclease an attractive target to improve the cytotoxicity and efficacy of platinum compounds including cisplatin and carboplatin. Metformin offers therapeutic potential against a variety of cancers when combined with other agents. However, whether metformin improves the radiosensitizing effects of platinum compounds still remains elusive in human lung cancer cells. In this study, we treated human NSCLC cells having different sensitivities to cisplatin with metformin alone or in combination with cisplatin and investigated the radiosensitivity as well as the underlying molecular mechanism.

## Results

### Effect of metformin and cisplatin on short-term NSCLC cell proliferation

**Figure 1:**
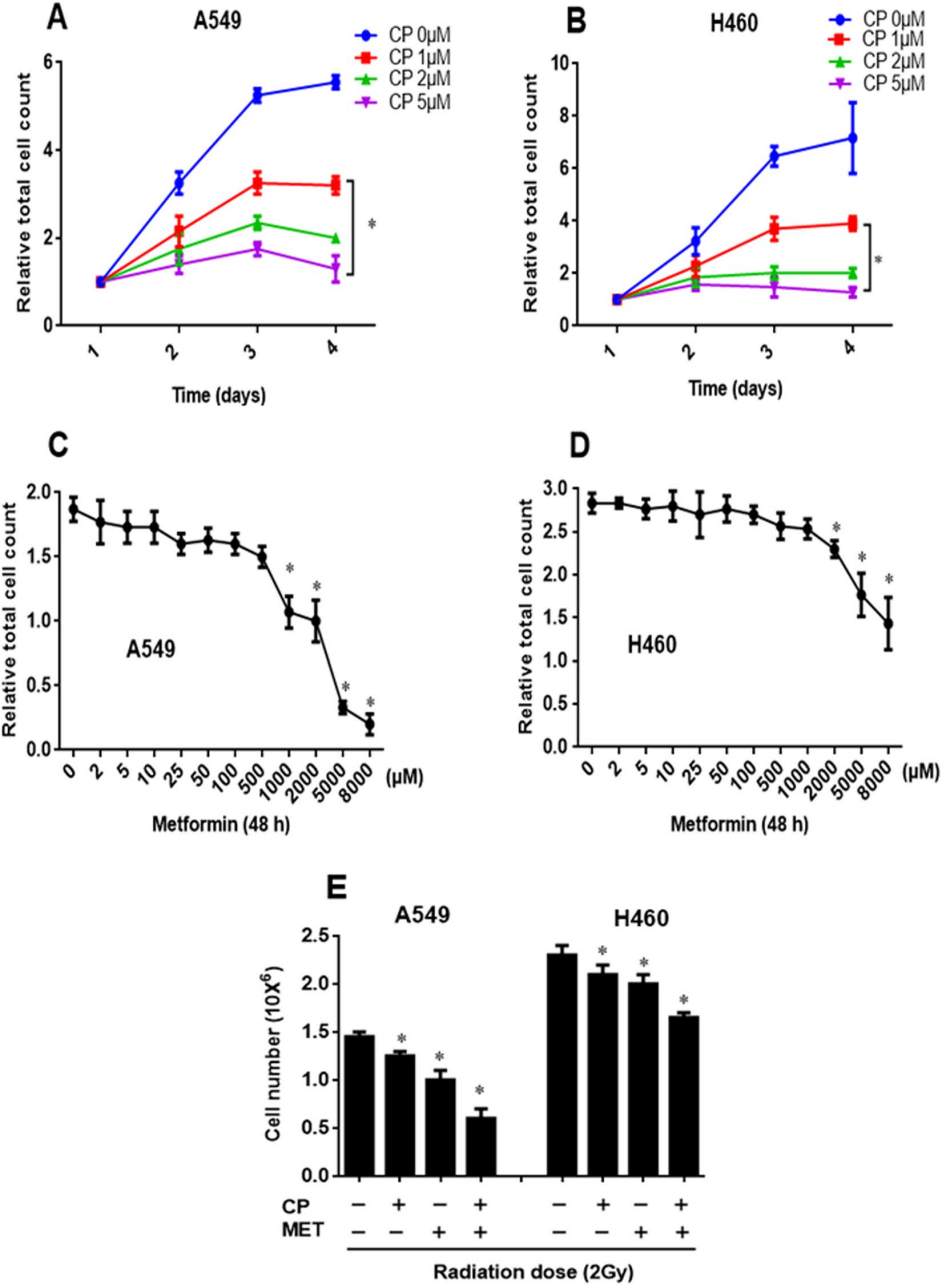
Effect of cisplatin and metformin on human NSCLC cell proliferation. (**A**,**B**) A549 and H460 human NSCLC cells were exposed to increasing concentrations (0–5 µM) of cisplatin (CP) and counted at 24, 48 and 72 h after cisplatin treatment. Proliferation results of 3–4 independent experiments are shown. Statistically significant differences compared with corresponding control cells (not treated with cisplatin) are shown (*P < 0.05). (**C**,**D**) A549 and H460 human NSCLC cells were treated with increasing concentrations of metformin (2 µM–8 mM) and cells were counted 48 h later. Proliferation results of 3–4 independent experiments are shown. Statistically significant differences compared with corresponding control cells (not treated with metformin) and 48 h metformin treatment groups are shown (*P < 0.05). (**E**) A549 and H460 cells were pre-treated with 1 and 2 mM metformin (MET), respectively, for 24 h followed by cisplatin (CP, 1 µM) for 4 h and subjected to 2 Gy of radiation. Cells were counted 24 h after radiation. Metformin was present in the medium after radiation. Results of 3–4 independent experiments are shown. Statistically significant differences of drug treated cells from untreated control cells (**−**) are shown: *P < 0.05 vs. untreated respective controls.

First, the concentration and time-dependent effects of cisplatin on proliferation were examined in the NSCLC cell lines A549 and H460. Previous pharmacokinetic and pharmacodynamic studies suggest that a concentration of 1**–**5 μM cisplatin is the optimal steady-state plasma level in cancer patients^39^. Therefore, A549 and H460 cells were treated with three clinically relevant concentrations of cisplatin (1, 2 and 5 μM) for 24**–**72 h. In both cell lines, cisplatin showed a concentration and time-dependent reduction in cell proliferation (Fig. 1A,B). The IC50 concentration of cisplatin was required to achieve 50% of maximal growth inhibition after 72 h of exposure, and their standard errors were 0.82 ± 0.07 μM for A549 and 0.85 ± 0.06 μM for H460, and were therefore similar. In addition, inhibitory effects of metformin on cell proliferation were also examined in NSCLC cells. A549 and H460 cells were exposed to increasing concentrations of metformin (2 µM–8 mM) and the cell numbers were determined 48 h after metformin treatment. Metformin showed a concentration dependent inhibitory effect on cell proliferation in both cell lines (up to 60–70% and 45–50% of control in A549 and H460 cells, respectively), as shown in Fig. 1C and D. At 1 mM concentration of metformin, the number of A549 cells was markedly reduced by more than 50% after 48 h. At a concentration of 2 mM, metformin showed even smaller decrease in H460 cell proliferation of 28% after 48 h. According to the different sensitivities of these cells to metformin, a concentration of 1 mM was used in the more metformin sensitive cell line A549 and of 2 mM in the less sensitive cell line H460 in all combined experimental settings to test combination effects of metformin with cisplatin on the radiation response.

Furthermore, both cell lines were also stimulated with metformin alone or in combination with cisplatin before radiation as indicated in Fig. 1E. In A549 cells, metformin or cisplatin combined with radiation resulted in a 10–15% and 30–35% reduction in cell numbers, respectively, as compared to only irradiated cells. The combination of metformin and cisplatin with radiation resulted in a further decrease in cell proliferation by about 55–60%, which was significantly (p < 0.05) stronger compared with each drug alone. In H460 cells, we did not observe a significant decrease in cell proliferation at 1 mM metformin concentration either alone or in combination with radiation compared to the respective controls (data not shown). However, 2 mM of metformin combined with radiation led to a reduction in cell number that was even stronger than the cisplatin-radiation treatment. The combination of metformin or cisplatin with radiation resulted in 10–15% and 15–20% reduction, respectively, which was even further decreased by 30–35% when both substances were given concurrently before radiation (Fig. 1E). These results suggest that combining metformin and cisplatin with radiation is more effective and show greater anti-proliferative effects compared with each drug alone *in vitro* in NSCLC cell lines.

### Metformin has no effect on radiation induced apoptosis in NSCLC cells

**Figure 2:**
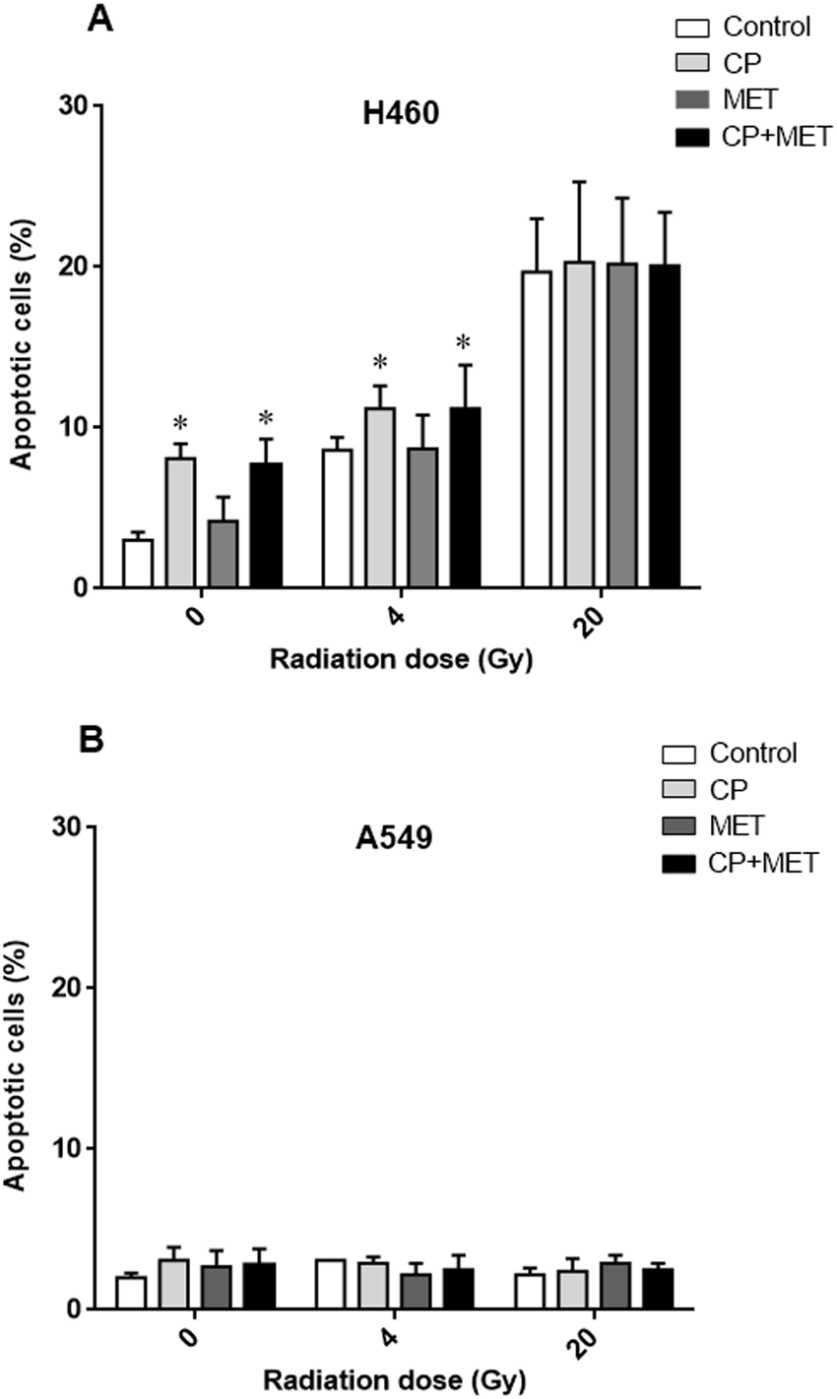
Effect of metformin (MET) on radiation-induced apoptosis in NSCLC cells. Cells were treated with metformin (1 mM for A549 and 2 mM for H460) for 24 h, subsequently cisplatin (CP, 1 µM) was added and cells were irradiated with 0 Gy, 4 Gy and 20 Gy at 4 h thereafter. Apoptotic fraction at 48 h after irradiation was determined by active caspase-3 assay. Data represent the mean ± SEM from 4 independent experiments. *p < 0.05 compared to non-treated cells.

Apoptotic cell death was measured by caspase-3 assay in NSCLC cells. Both cell lines were pre-treated with metformin (A549 cells with 1 mM, H460 cells with 2 mM) for 24 h followed by cisplatin treatment (1 µM) for another 4 h. Subsequently cells were irradiated to 4 Gy and 20 Gy and the apoptotic fraction was determined by active caspase-3 assay 48 h after irradiation (Fig. 2). Apoptotic response of H460 to radiation was different in both cell lines with a significant dose dependence of apoptotic cell death ranging from 3.0% ± 0.5% of sham-irradiated controls to 8.6% ± 0.8% and 19.7% ± 3.3% after irradiation with 4 Gy and 20 Gy, respectively (p < 0.0001, ANOVA F-test, Fig. 2A). In comparison, A549 cells were highly resistant to apoptosis with <3% apoptotic cell death, irrespective of the treatment and there was no significant dependence of apoptosis on radiation dose, cisplatin or metformin exposure (p > 0.20, ANOVA F-test, Fig. 2B). Cisplatin increased apoptosis in the apoptosis competent cell line H460 slightly by 2.38% ± 0.58% (p < 0.0002, ANOVA F-test), while metformin had no effect on apoptosis (p = 0.73, ANOVA F-test). There was no interaction effect between radiation dose and exposure with cisplatin or metformin in H460 cells (p = 0.22, ANOVA F-test). Overall, apoptotic cell death is not an important mechanism for the radiosensitizing effects of metformin.

### Metformin improves the radiosensitizing effect of cisplatin in NSCLC cells in the long term clonogenic assay

**Figure 3:**
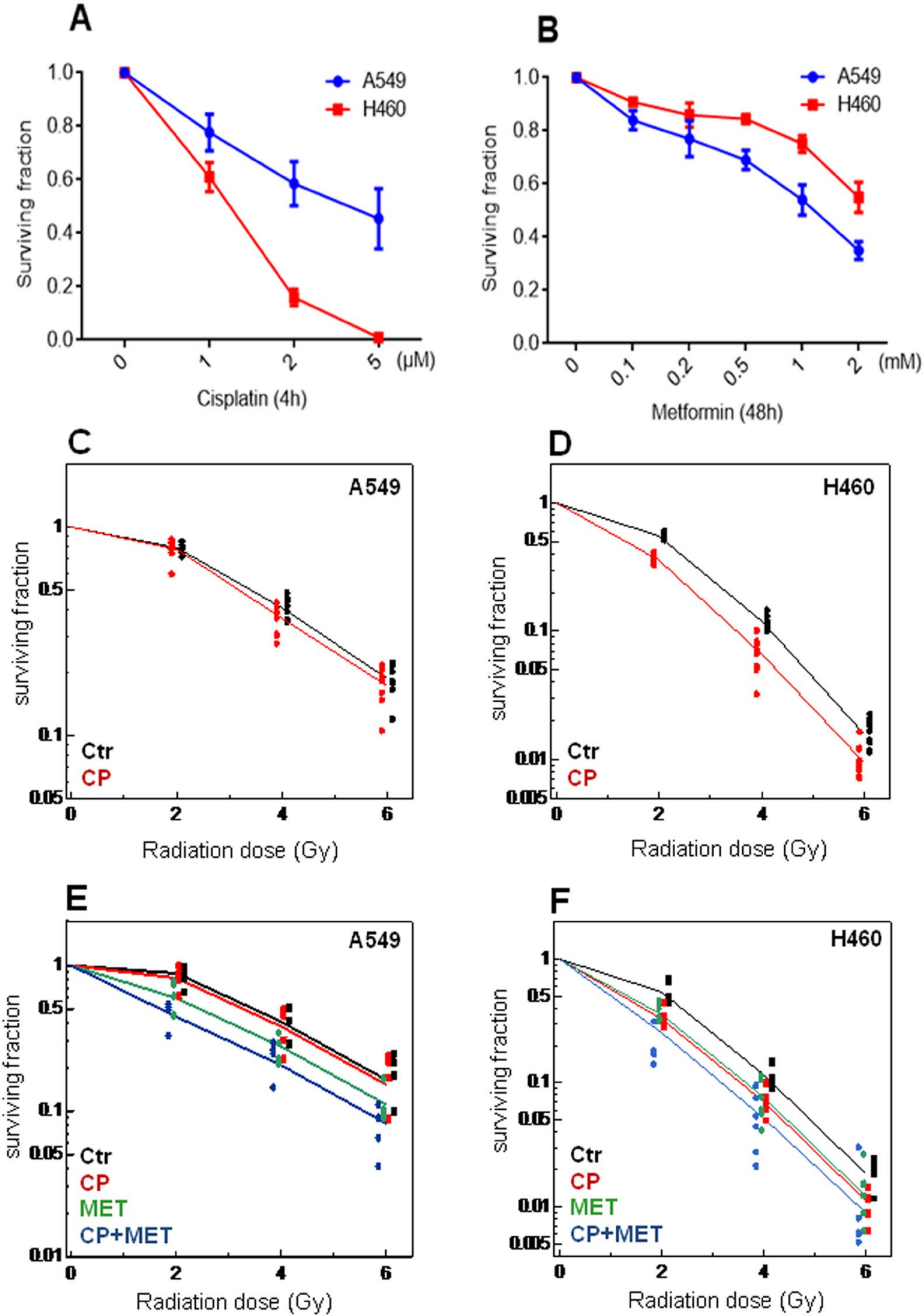
Effect of cisplatin and metformin on clonogenic survival and radiosensitization of human NSCLC cells. (**A**) Cisplatin reduced clonogenic survival of NSCLC cells. A549 and H460 cells were treated with increasing concentrations of cisplatin (0–5 µM) for 4 h and clonogenic survival was determined. Average results of 2–3 independent experiments are shown. (**B**) Metformin reduced clonogenic survival of NSCLC cells. A549 and H460 cells were treated with increasing concentrations of metformin (0–2 mM) for 48 h and clonogenic survival was determined. Average results of 2–3 independent experiments are shown. (**C**,**D**) Survival curves for cells treated with cisplatin (red dots) versus untreated cells (black dots) after irradiation. Both cell lines were pre-treated with cisplatin (CP, 1 µM) for 4 h and subsequently exposed to radiation doses of 0–6 Gy. Clonogenic survival was determined and data at doses between 2 Gy and 6 Gy were fitted to a general linear model. Average results of 5–6 independent experiments are shown. (**E**,**F**) Metformin enhances the radiosensitizing effects of cisplatin in NSCLC cells. A549 and H460 cells were first treated with 1 mM and 2 mM metformin (MET), respectively, for 48 h followed by cisplatin (CP) treatment (1 µM) for 4 h. Cells were subsequently subjected to 0–6 Gy of radiation. Clonogenic survival was determined and data of 5–6 independent experiments are shown.

To determine the effect of cisplatin on clonogenic survival of NSCLC cells, H460 and A549 cells were exposed to cisplatin (1, 2 and 5 µM) for 4 h and clonogenic cell survival assays were performed as described under methods. Cisplatin reduced colony formation in a dose dependent manner in both cell lines (Fig. 3A). H460 cells showed a greater sensitivity to cisplatin with 41% inhibition of colony formation at 1 µM cisplatin and 80% inhibition at 2 µM, compared with 23% and 32%, respectively, for A549 cells. Next, we assessed the ability of metformin to inhibit colony formation of A549 and H460 cells. Both cell lines were treated with increasing concentrations of metformin (0.1, 0.2, 0.5, 1 and 2 mM) and colony formation was markedly reduced by metformin (0.5 mM and higher concentrations) in both cell lines (Fig. 3B). In contrast to the cisplatin effects, A549 cells showed a greater sensitivity to metformin with 31% inhibition of colony formation at 0.5 mM metformin, 46% inhibition at 1 mM and 65% inhibition at 2 mM metformin compared with 16%, 25% and 46%, respectively, for H460 cells. At a concentration of 1 µM, cisplatin showed detectable anti-proliferative and anti-clonogenic effects in A549 and H460 cells (Figs 1A,B and 3A) but was below the half maximal inhibitory concentration in both cell lines. The subsequent experiments were performed at this concentration. As shown in Fig. 3C and D, cisplatin significantly increased the radiosensitivity of H460 cells (p < 0.0001, ANOVA F-test) but not of A549 cells (p = 0.064, ANOVA F-test). To test, whether metformin could further radiosensitize these cells exposed to cisplatin, A549 and H460 cell lines were stimulated with 1 mM and 2 mM of metformin for 48 h, respectively, before cisplatin treatment (1 µM) and clonogenic survival was examined after irradiation to 0, 2, 4 and 6 Gy. Treatment with metformin alone significantly increased the radiosensitivity of both cell lines (p < 0.0001, ANOVA F-test) with and without cisplatin exposure compared with respective controls treated at the same radiation doses but without metformin. The co-treatment of metformin with cisplatin showed a supra-additive effect on the radiosensitivity of A549 cells (p = 0.018 for the interaction effect between cisplatin and metformin), whereas metformin when combined with cisplatin displayed an additive effect on the radiosensitivity of H460 cells (p = 0.71 for the interaction effect between cisplatin and metformin, Fig. 3E,F).

### Effects of metformin on radiation-induced DNA damage repair foci in NSCLC cells

**Figure 4:**
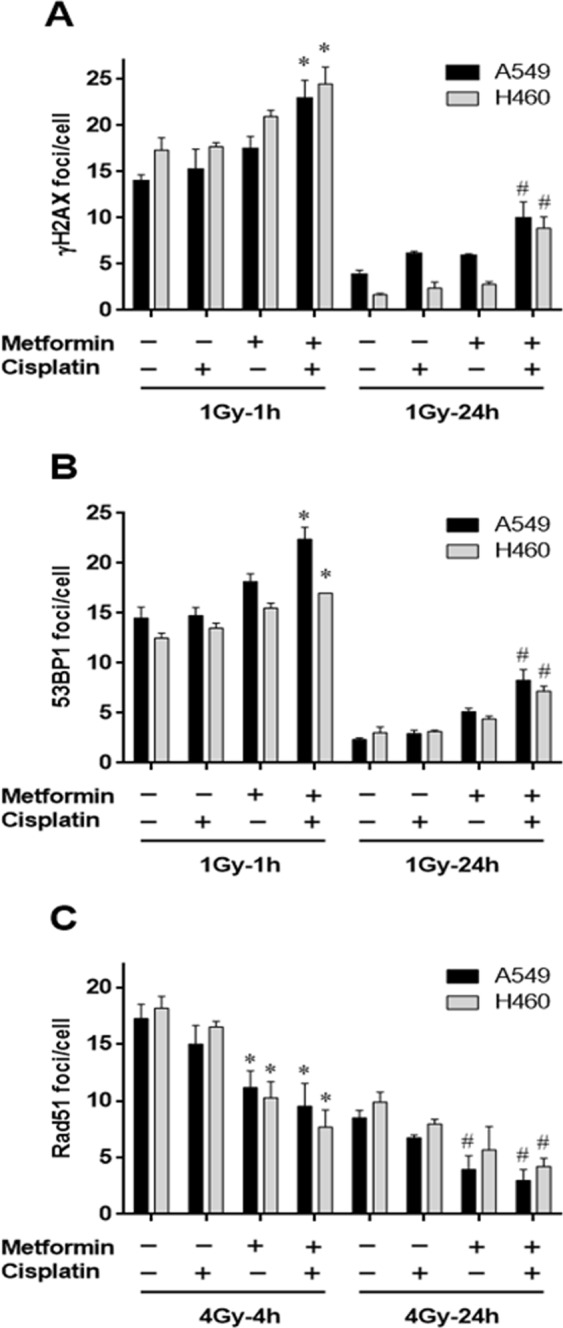
Effect of cisplatin and metformin on radiation-induced DNA damage in NSCLC cells. Induction of γ-H2AX and 53BP1 foci by metformin and cisplatin in irradiated cells (**A**,**B**). A549 or H460 cells were treated with 1 mM and 2 mM metformin for 24 h, respectively, followed by cisplatin (1 µM) for another 4 h as indicated. After irradiation (1 Gy), cells were fixed and stained for γ-H2AX (**A**) and 53BP1 (**B**). The numbers of γ-H2AX and 53BP1 were visually counted for each cell and the average number of foci per cell was recorded. Data represent the mean number of foci per cell in at least 50 cells in 3–4 independent experiments. (*P < 0.05 compared to cisplatin plus 1Gy-1h or 1Gy-1h alone; ^#^P < 0.05 compared to cisplatin plus 1Gy-24h or 1Gy-24h alone). (**C**) Induction of Rad51 foci by metformin and cisplatin in irradiated cells. A549 or H460 cells were pre-treated with metformin alone or in combination with cisplatin as indicated. After exposure to radiation (4 Gy), cells were fixed and stained for Rad51. Data represent the mean number of foci per cell in at least 50 cells in 3–4 independent experiments. (*P < 0.05 compared to cisplatin plus 4Gy-4h or 4Gy-4h alone; ^#^P < 0.05 compared to cisplatin plus 4Gy-24h or 4Gy-24h alone).

To test whether metformin-enhanced radiosensitizing action of cisplatin in A549 and H460 cell lines is associated with inhibition of the DNA repair process, we investigated the effect of metformin on the DNA damage and repair by immunofluorescence microscopy for γH2AX and 53BP, established markers of DNA-double-strand breaks (DNA-DSB) and Rad51, a marker of homologous recombination. The γH2AX foci formation was examined at 1 h and 24 h after irradiation as a measure for initial and residual DNA-DSB, respectively. The quantitative results are shown in Fig. 4 and representative images in Supplementary Figure S1. Cisplatin combined with radiation showed no increase in initial γH2AX foci levels in both cell lines. Metformin combined with radiation resulted in a slight increase in initial γH2AX foci in both cell lines compared with only irradiated cells. However, the combination of cisplatin and metformin with radiation led to an increased number of initial foci in both cell lines (1.6-fold in A549 and 1.4-fold in H460 cells) compared with each compound alone. The number of γH2AX foci markedly decreased within 24 h after radiation as compared to the initial foci level, indicating the DNA repair. The combination of either cisplatin or metformin with radiation yielded a slight increase in residual γH2AX foci in both cell lines. Co-treatment of metformin with cisplatin applied before radiation showed significantly higher (p < 0.05) numbers of residual γH2AX foci in these cells (2.5-fold in A549 and 3.7-fold in H460) compared to the residual number of γH2AX foci for only irradiated cells (Fig. 4A). These observations were further confirmed by evaluating the effect of metformin together with cisplatin on 53BP1 foci formation in irradiated NSCLC cells. Similar to the data presented in Fig. 4A, cisplatin in combination with radiation also showed no increase in the initial as well as the residual numbers of 53BP1 foci as compared with irradiation alone. Metformin alone showed a slight increase in radiation-induced initial and residual 53BP1 foci numbers in each cell line. However, as shown for γH2AX foci, the combined metformin and cisplatin treatment also yielded significantly higher numbers (p < 0.05) of initial as well as residual 53BP1 foci in A549 and H460 cells compared with either treatment alone (Fig. 4B). Next, we assessed the effect of metformin and cisplatin on DNA damage repair by examining the Rad51 foci formation (Fig. 4C). Cisplatin caused a slight but insignificant decrease in radiation-induced initial and residual Rad51 foci numbers compared with only irradiated cells. Metformin showed a marked reduction in initial as well as the residual Rad51 foci numbers in both cell lines, which were further significantly reduced (p < 0.05) when both drugs were simultaneously used before radiation as compared with each treatment alone. To exclude that the reduction of Rad51 foci by metformin is not due to changes in cell cycle, A549 and H460 cells were exposed to increasing concentrations of metformin (0.002–5 mM) for different times periods (24 and 48 h) and cell cycle phases were evaluated. Overall, cell cycle profiles did not show significant differences between metformin treated and untreated cells (data not shown). These data show that increased radiosensitivity of NSCLC cancer cell lines after metformin and cisplatin is paralleled by altered kinetics of γH2AX, 53BP1 and Rad51 foci.

### Effects of metformin on cisplatin-GpG DNA adduct formation in NSCLC cells

**Figure 5:**
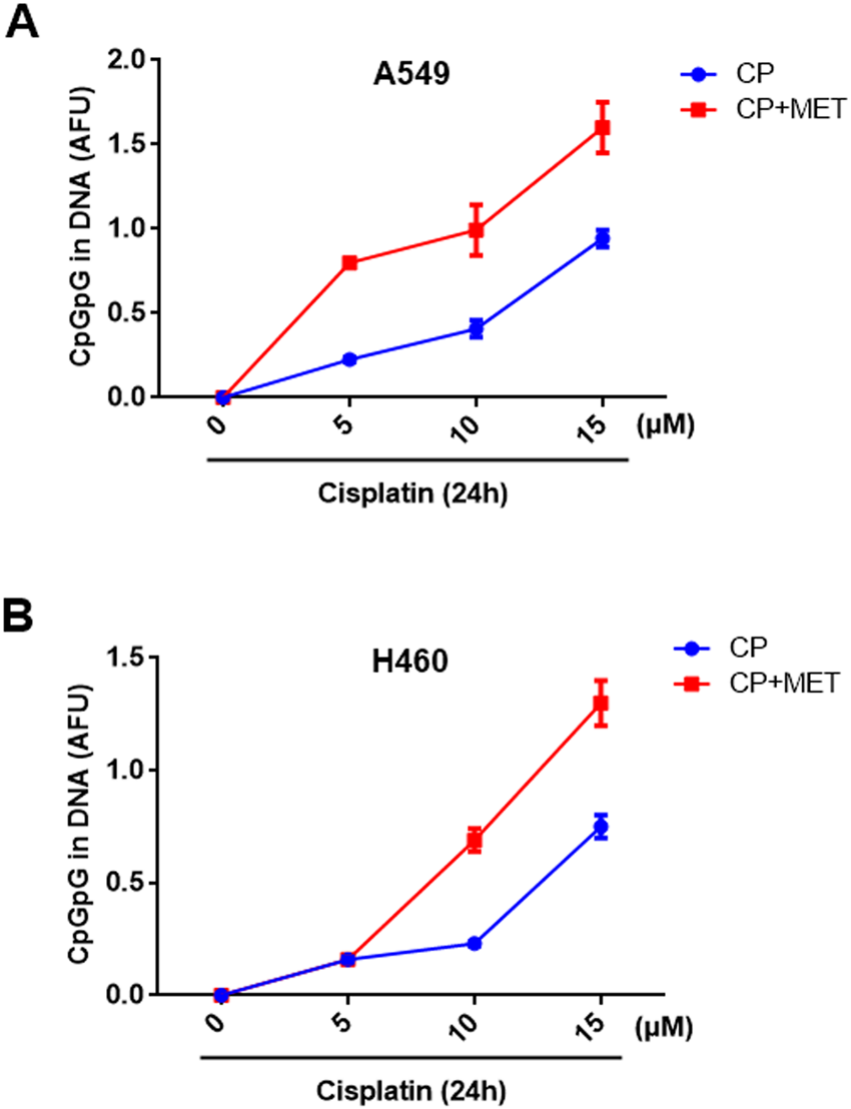
Effect of metformin on cisplatin-DNA adducts formation in NSCLC cells. Metformin (MET) enhances cisplatin**-**DNA adducts formation. A549 (**A**) and H460 (**B**) cells were treated with or without 1 mM and 2 mM MET for 24 h, respectively. Cells were then exposed with the indicated concentrations of cisplatin (CP) and DNA adducts were quantified 24 h later. Data are presented as the mean AFU ± 95% confidence interval from 2–3 independent experiments.

To assess the levels of cisplatin**-**DNA adducts, cisplatin**-**(GpG) intrastrand cross-links in cellular DNA were examined by quantitative immunocytological measurements using a monoclonal antibody. Pre-exposure of A549 cells with metformin at 1 mM over 20 h before and during cisplatin (5, 10 and 15 µM) over 24 h further enhanced the cisplatin-DNA adducts formation as compared with cisplatin treatment alone (Fig. 5A). Analysing the data with a linear quadratic covariance model, the adduct level was dependent on the square of the cisplatin concentration with a coefficient of 0.0035 ± 0.0005 fluorescence intensity units/μM^2^ (p < 0.0001, ANOVA F-test) and metformin increased this cisplatin dependence by an interaction effect of 0.0030 ± 0.0008 fluorescence intensity units/μM^2^ (p < 0.0012, ANOVA F-test). In H460 cells, the effect of pre-exposure of metformin at 2 mM before and during cisplatin (5, 10 and 15 µM) on cisplatin-DNA adduct formation is shown in Fig. 5B. The adduct level was again dependent upon the square of the cisplatin concentration with a coefficient of 0.0040 ± 0.0006 fluorescence intensity units/μM^2^ (p < 0.0001, ANOVA F-test). However, cisplatin concentrations from 10–15 µM showed a massive additive cisplatin concentration-independent effect on adduct formation with an estimate of 0.58 ± 0.10 fluorescence intensity units (p = 0.0001, ANOVA F-test).

### Effects of metformin and cisplatin on the expression of DNA repair proteins in NSCLC cells

**Figure 6:**
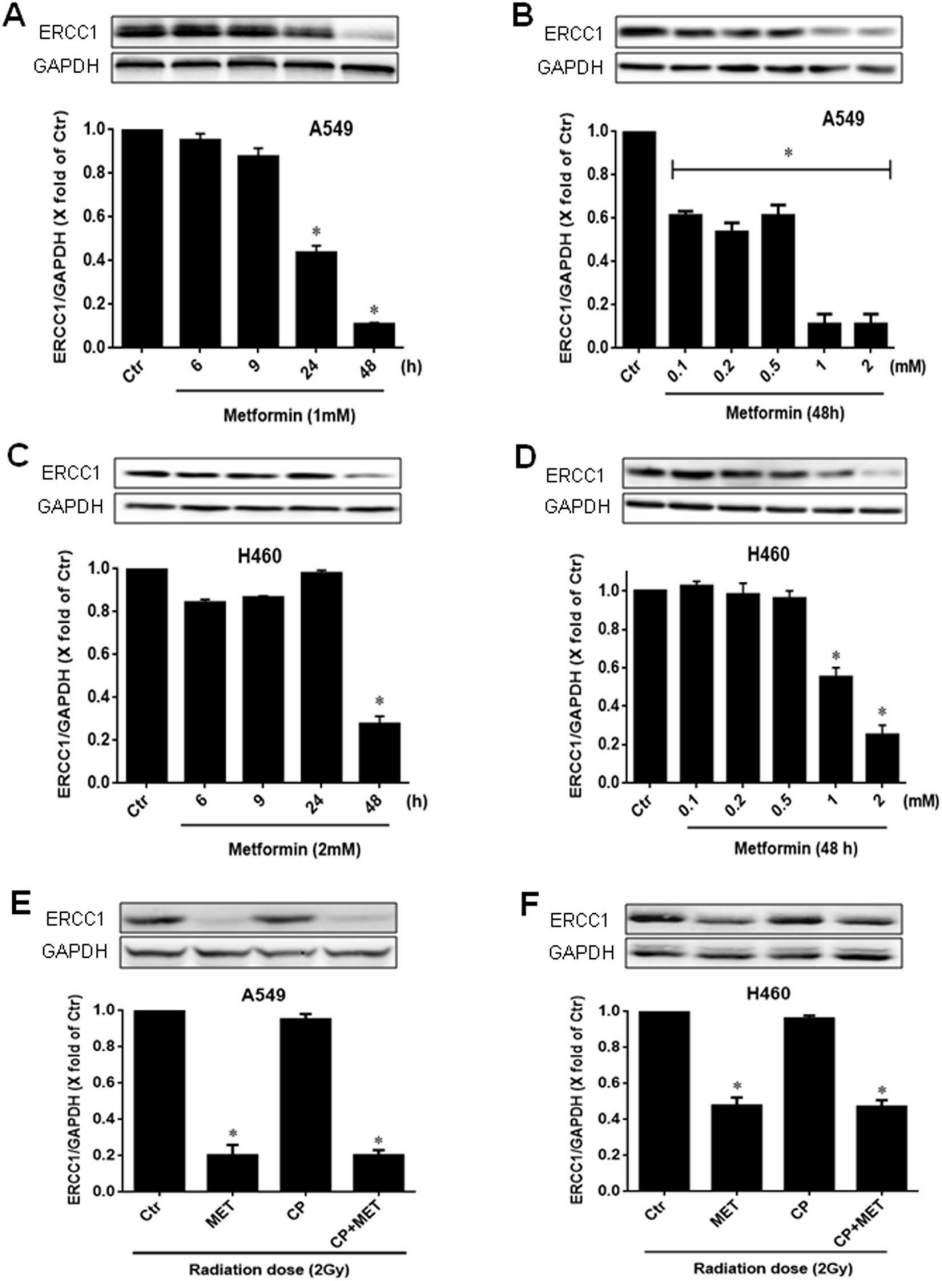
Effect of metformin on ERCC1 expression in NSCLC cells. A549 and H460 cells were treated with 1 mM and 2 mM of metformin, respectively, for 0–48 h, lysed and probed with anti-ERCC1 and anti-GAPDH antibodies. Representative immunoblots (top) and the corresponding densitometric quantification (bottom) from 3–4 independent experiments are shown. *P < 0.05 compared to untreated cells (Ctr) (**A**,**C**). A549 and H460 cells were treated with metformin (0.1–2 mM) for 48 h, lysed and probed with anti-ERCC1 and anti-GAPDH antibodies. Representative immunoblots (top) and the corresponding densitometric quantification (bottom) from 3–4 independent experiments are shown. *P < 0.05 compared to untreated cells (Ctr) (**B**,**D**). A549 and H460 cells were pre-treated with 1 mM and 2 mM of metformin (MET), respectively, for 24 h with or without CP (1 µM) for further 4 h, followed by exposure to 2 Gy of radiation. Cells were lysed 24 h after radiation and probed with the indicated antibodies. Representative immunoblots (top) and the corresponding densitometric quantification (bottom) from 3–4 independent experiments are shown. *P < 0.05 compared to only irradiated cells (Ctr) (**E**,**F**).

The primary pathway that is mainly involved in the repair of cisplatin-DNA adducts is the nucleotide excision repair pathway (NER). Thus, we next examined the effect of metformin on the expression of ERCC1, a key component of the NER pathway. To do this, A549 and H460 cells were treated with increasing concentrations of metformin (0.1, 0.2, 0.5, 1 and 2 mM) for various times (6, 9, 24 and 48 h) as indicated. Western blot analysis showed a concentration and time-dependent decrease in ERCC1 protein expression after metformin treatment. A549 and H460 cells treated with 1 mM and 2 mM metformin, respectively, for 6 h and 9 h did not show any significant ERCC1 reduction. However, 24 h and 48 h metformin showed a marked reduction in ERCC1 protein expression in A549, whereas, ERCC1 protein expression was decreased after 48 h in H460 cells (Fig. 6A,C), indicating an inhibitory action of metformin in the NER pathway. The effect greatly depends on the concentration of metformin exposure in both cell lines, with maximal effects shown after 1 mM in A549 and 2 mM in H460 cells after 48 h, respectively (Fig. 6B,D). We also examined the effects of metformin and cisplatin together with radiation on ERCC1 expression in both cell lines. As shown in Fig. 6E,F, metformin in combination with radiation also significantly (p < 0.05) inhibited the ERCC1 expression compared to their respective controls in both cell lines (radiation alone). The combination of metformin and cisplatin with radiation treatment caused no further reduction in ERCC1 expression as compared with metformin and radiation alone. Moreover, cisplatin alone in combination with radiation also showed no discernable effect on ERCC1 protein expression under the same experimental settings in both cell lines (Fig. 6E,F).

### Effect of AMPK inhibition on metformin-enhanced radiosensitivity of NSCLC cells

**Figure 7:**
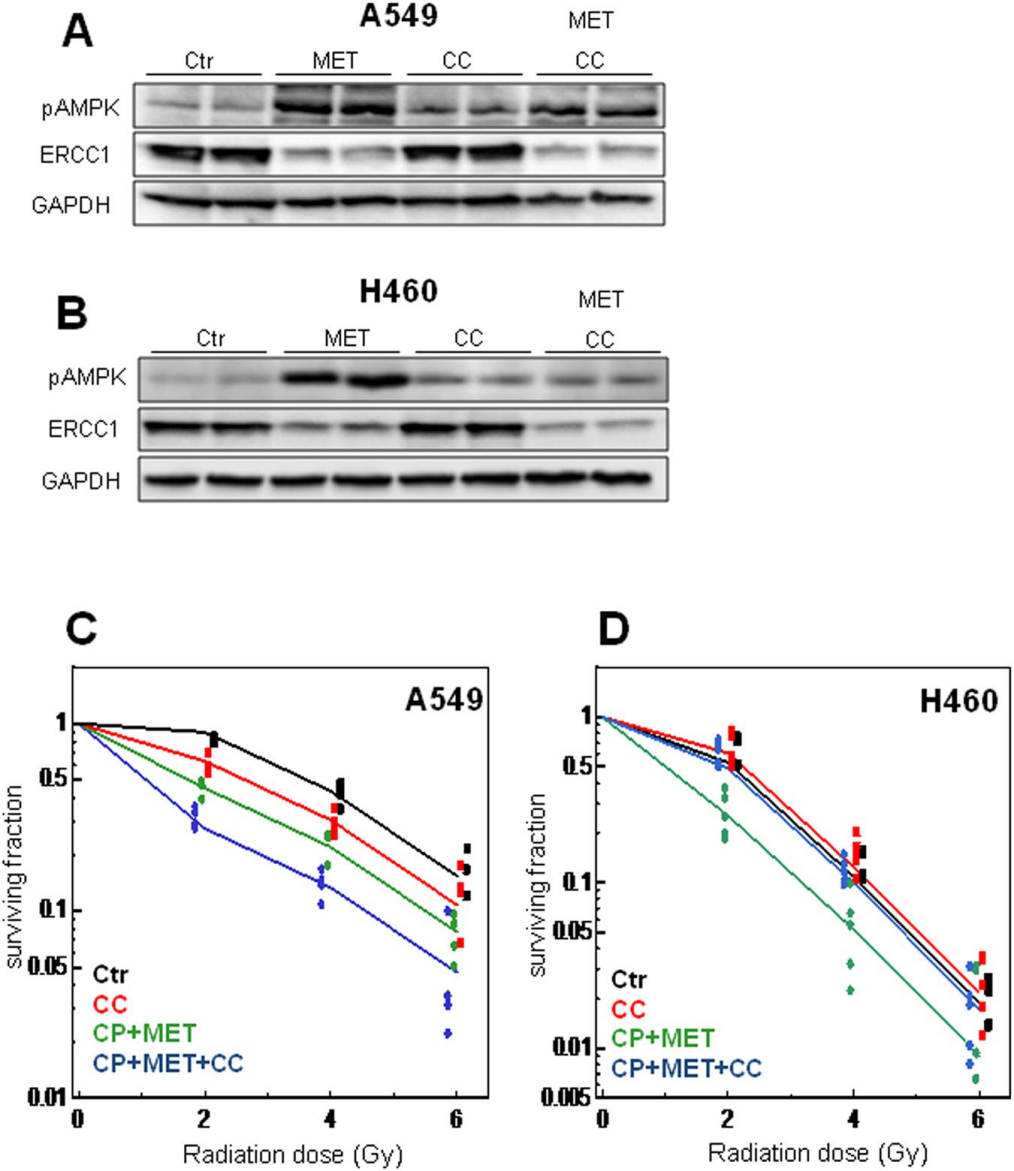
Effect of AMPK inhibition on metformin**-**enhanced radiosensitizing effect of cisplatin in NSCLC cells. A549 or H460 cells were pre-incubated with 3 µM of Compound-C (CC) for 2 h followed by 1 mM and 2 mM metformin (MET), respectively, for further 48 h. Cells were lysed and probed with anti**-**pAMPK (Thr 172), anti-ERCC1 and anti-GAPDH antibodies. Representative immunoblots of phosphorylated AMPK and ERCC1 expression in A549 cells (**A**) and H460 cells (**B**) are shown. A549 or H460 cells were pre-stimulated with 3 µM of Compound**-**C (CC) for 2 h followed by 1 mM and 2 mM of metformin (MET) treatments, respectively, for further 48 h. Cells were subsequently stimulated with 1 µM cisplatin for 4 h, washed with PBS and subjected to 0, 2, 4 or 6 Gy radiation and clonogenic assay was determined in A549 (**C**) and H460 cells (**D**). Results of 5–6 independent experiments are shown.

The best known mechanism of metformin action is to activate adenosine activated protein kinase (AMPK) through the phosphorylation of AMPKα at Thr-172 in different experimental models^30^. Therefore, to investigate whether metformin activates AMPK in NSCLC cells, the level of AMPK phosphorylation was analyzed after metformin treatment. A549 and H460 cells were treated with 1 mM and 2 mM metformin, respectively and AMPK phosphorylation was checked after 48 h by western blot analysis. As shown in Fig. 7A and B, the metformin treatment resulted in a strong increase in AMPK phosphorylation in both cell lines. In order to determine the role of AMPK in mediating metformin-enhanced radiosensitivity of NSCLC cells we used Compound-C (3 µM), an AMPK inhibitor, which effectively suppressed the metformin-induced AMPK phosphorylation as compared with cells treated only with metformin. Furthermore, the metformin-suppressed ERCC1 expression was retained on same level in the presence of Compound-C, indicating an AMPK independent action of metformin on ERCC1 protein expression. We next sought to investigate whether AMPK inhibition could abolish the radiosensitizing effects of metformin. To this end, AMPK was inhibited with Compound-C and clonogenic survival of irradiated cells was examined after a combined treatment with metformin and cisplatin. AMPK inhibition did not abolish the radiosensitizing ability of metformin in A549 cells. We observed, that Compound-C significantly increased (p < 0.0001, ANOVA) the radiosensitivity of A549 cells with or without cisplatin and metformin exposure in an additive manner (Fig. 7C), suggesting the involvement of AMPK pathway in the radiosensitivity of these cells. The interaction effect between Compound-C exposure and cisplatin + metformin exposure was not significant (p = 0.37, ANOVA F-test). On the other hand, AMPK inhibition by Compound-C completely abolished the significant ability of the metformin + cisplatin combination to enhance the radiosensitivity of H460 cells (p < 0.0001 for the cisplatin + metformin effect, ANOVA-F-test, Fig. 7D). There was a significant negative interaction effect between Compound-C exposure and cisplatin + metformin exposure (p = 0.0009, ANOVA F-test), indicating that the radiosensitizing effect of the metformin + cisplatin combination is AMPK dependent in H460 cells.

## Discussion

The presented data show that combined treatment with cisplatin and metformin with radiation enhanced the radiosensitizing effect of cisplatin compared with each treatment alone. Multiple epidemiological studies documented the preventive and therapeutic effects of metformin for various human cancers^40–42^. A direct association has also been observed between metformin treatment and the reduction of mortality rate and cancer incidence in patients with type 2 diabetes^43^. In this context, several experimental investigations concentrated on the potential role of metformin as a radiosensitizer for different malignancies^30,44–46^. Metformin enhanced the radiosensitivity of breast cancer and fibrosarcoma cells and was found to be highly effective to eradicate radioresistant tumors, suggesting that metformin is a useful therapeutic agent to enhance the efficacy of radiotherapy for such tumors. AMPK and mammalian target of rapamycin (mTOR) are important signalling proteins for the cytotoxic and radiosensitizing action of metformin both *in vitro* and *in vivo*. Storozhuk *et al*. have clearly documented that activation of AMPK pathway is correlated with radiosensitizing effects of metformin in lung cancer cells^30^. As a radiosensitizer, metformin has also been very effective in the treatment of many other types of cancers including prostate and head and neck cancers^29,47,48^. Apoptosis and DNA damage repair have also been discussed as the mechanisms underlying the radiosensitizing effect of metformin^30^. In our study, metformin has no significant effect on radiation induced apoptosis in both cell lines. Thus, the radiosensitizing effect of metformin occurred mainly through increased DNA damage by inhibiting DNA repair proteins.

Cisplatin is one of the most commonly used chemotherapy drugs to enhance the radiosensitivity of multiple tumors^49–52^. However in this study, we show that cisplatin radiosensitizes H460 cells but not A549 cells, indicating that the enhancement of radiosensitivity by cisplatin is not a general phenomenon and is restricted to certain cell types, a finding that is in accordance with previous reports^53,54^. Toulany *et al*. suggested that the lack of radiosensitizing action of cisplatin in A549 cells is associated with cisplatin-induced activation of ataxia-telangiectasia mutated (ATM) and autophagy that can act cytoprotective. Other studies have reported several mechanisms responsible for the differential effects of the cisplatin sensitivity in various tumors^55,56^. Among them, limiting the formation of cisplatin-DNA adducts, enhanced repair of these adducts and/or increased tolerance of the resulting DNA damage appears to be of particular importance^5,6,57^. In the present study, we observed the dose and time dependent inhibitory effects of metformin on NSCLC cell proliferation that was further enhanced when combined with cisplatin. Furthermore, the stimulation of A549 (cisplatin resistant) and H460 cells (cisplatin sensitive) with metformin also reduced the clonogenic survival of both cell lines. The combined treatment with cisplatin and metformin with radiation led to a supra-additive (A549) and additive (H460) effect on the radiosensitivity in the clonogenic assay. Previous reports have also reported that metformin could serve as a chemosensitizer for cisplatin-based regimens and would be effective for different cancer cells. Using *in vitro* and *in vivo* models, Qi *et al*. have recently documented that metformin sensitizes the response of oral squamous cell carcinoma to cisplatin^58^. Metformin synergistically elevated the cytotoxic effect of cisplatin and reversed the chemoresistance through inhibiting NF-κB/HIF-1α signal axis. In the present study, metformin alone showed marked anti-cancer effects, perhaps because of the relatively higher concentration used in our experiments. Several studies have demonstrated that significant inhibition of cell growth and tumor development could occur at higher doses of metformin in a variety of tumor cell types^44,59,60^. In fact, numerous studies have shown that metformin may potentiate the efficacy of multiple anti-cancer drugs including cisplatin^33–37^. The anti-cancer activity of cisplatin is primarily based on its binding with the cellular DNA and the formation of cisplatin-adducts^61^, which leads to apoptosis and cell death through impairing multiple cellular processes. These adducts are removed mainly by the nucleotide excision repair (NER) pathway from the cellular DNA^62^. In the present study, we confirmed the dose dependent increase of cisplatin-DNA adducts following cisplatin stimulation in both cell lines. Interestingly, cisplatin exposure led to higher levels of DNA adducts when combined with metformin as compared with the cisplatin treatment alone. However, the effect of metformin on cisplatin-DNA adducts was more pronounced in A549 in comparison to H460 cells and led to a differing adduct response relation on the cisplatin concentration in A549 and H460 cells. These data indicate that metformin may increase the binding of cisplatin to cellular DNA or inhibit the repair of the cisplatin-DNA adducts. The excision repair cross complementing-group 1 (ERCC1) is an essential protein of the NER pathway that repairs multiple DNA adducts and is associated with cellular resistance to platinum-based chemotherapy^7,8,63^. Our results also showed a strong reduction in the expression of ERCC1 protein after metformin treatment in both cell lines, which indicates a possible involvement of the NER pathway in the radio-enhancement effect of the combined cisplatin and metformin treatment observed in the present study. In fact, the levels of metformin-enhanced cisplatin adducts observed in this study, paralleled well with the enhanced effect of cisplatin on radiation sensitivity of NSCLC cells. In addition, a higher inhibitory effect of metformin on ERCC1 expression in A549 cells, in comparison to H460 cells may in part explain the more pronounced combination effect of metformin in A549 with cisplatin and radiation treatment. These results are in agreement with previous studies suggesting that ERCC1 gene targeting can enhance cisplatin potency^13,15,16,64^.

The most effective damage induced by ionizing radiation are DNA double-strand breaks which are repaired by non-homologous and homologous recombination repair. In the present study we found that metformin combined with cisplatin leads to an increase in radiation**-**induced initial and residual γH2AX foci, a phosphorylation product of H2AX by PIKK family proteins including ATM, as compared with cisplatin treatment alone. γH2AX foci are sensors of DNA double-strand breaks. Thus, metformin pre-incubation leads to a larger prevalence of radiation-induced γH2AX foci at later times after irradiation, possibly related to an increased remaining DNA damage. To further confirm this observation, we examined the effects of metformin on radiation-induced 53BP1 foci formation, which is another well-known sensor protein of DNA damage. In the analysis of 53BP1 foci formation after irradiation, cisplatin and metformin exposure showed similar effects as that of γH2AX foci. We also observed that metformin alone or in combination with cisplatin, significantly decreased radiation-induced initial and residual Rad51 foci formation, a marker for homologous recombination, compared with cisplatin treatment alone. Therefore, multiple molecular mechanisms leading to increased cisplatin-DNA adduct formation and increased radiation response can play a role.

It is well documented that metformin can signal through activation of AMPK by increasing the phosphorylation of Thr172 on its α-catalytic subunit^65^. In this respect, we examined the effects of metformin on AMPK phosphorylation in NSCLC cells. The treatment with metformin led to a strong increase in AMPK phosphorylation that was significantly suppressed by the AMPK inhibitor, Compound-C in both cell lines. Here we showed that Compound-C alone had no effect on the radiosensitivity of cisplatin-sensitive H460 cells and completely abolished the radiosensitizing effect of metformin, indicating that a combined treatment with cisplatin and metformin exerts its action through AMPK pathway in H460. In contrast, pharmacological inhibition of AMPK failed to abrogate but increased the radiosensitizing effect of metformin in cisplatin-resistant A549 cells. A previous study has documented similar differing effects of Compound-C combined with cisplatin alone on the radiation sensitivity of A549 and H460 cells^54^. The increased phosphorylation of cytoplasmic and nuclear ATM upon cisplatin exposure was found to be associated with less radiosensitization in A549. Less ATM phosphorylation was observed in H460 cells. The radiosensitizing effect of cisplatin was reverted by Compound-C in H460 cells, while in A549, without a radiosensitizing effect of cisplatin, this effect was increased after combined treatment with cisplatin and Compound-C. Therefore, the radiosensitizing effect of cisplatin alone might be AMPK dependent in H460 but not in A549. In addition, Liu *et al*.^66^ demonstrated that Compound-C can lead to cell death of cancer cells by multiple mechanisms, such as inhibition of proliferation and cell migration, increase of G2/M block, autophagy and apoptosis, all of which are independent of AMPK.

In conclusion, we demonstrated that metformin can enhance the sensitivity to a combined treatment of cisplatin and ionizing radiation in H460 and A549 NSCLC cells, with a higher effect in the A549 cell line that is less sensitized by cisplatin. This effect can be reverted by Compound-C in H460 cells but is increased in A549 cells. Retrospective cohort studies suggest, that metformin can improve the outcome of diabetic patients with locally advanced non-small cell lung cancer, treated with concurrent radiochemotherapy or of diabetic Stage IV NSCLC patients, treated with chemotherapy^67,68^. Prospective randomized trials are underway to test the targeted therapy or radiochemotherapy sensitizing effect of metformin in non-small cell lung cancers^69–71^. Thus, metformin is a promising candidate for a combination with concurrent first line radiation-chemotherapy in locally advanced lung cancer.

## Materials and Methods

### Cell lines

The human non-small cell lung carcinoma NSCLC cell lines A549 and H460 were obtained from American Type Culture Collection (ATCC, Rockville, MD, USA). The H460 cell line was maintained in RPMI-1640 (Invitrogen, Paisley, UK) supplemented with 10% FBS plus antibiotics. The A549 cell line was maintained in MEM (Invitrogen) supplemented with 15% FBS plus 1% non-essential amino acids and antibiotics. NSCLC cell lines were propagated in medium supplemented with 5 µg/ml plasmocin (InvivoGen, Toulouse, France) as a mycoplasma prophylaxis and were cultivated in an atmosphere consisting of 5% CO_2_ and 95% air at 37 °C. Both cell lines were usually re-thawed after 3 month in culture. Cells were irradiated using an X-ray machine RS320 (Xstrahl Ltd, Surrey, UK) at 300 kV, 10 mA, dose rate 0.9 Gy/min.

### Experimental protocols

All experiments were performed in complete cell culture medium containing FCS, unless otherwise stated. Stock solutions of cisplatin, metformin and Compound-C were prepared in water and added to the cells as indicated. When a combination of drugs was used, Compound-C was added 2 h before adding the metformin.

### Cell proliferation

NSCLC cells were seeded at a density of 4 × 10^5^ cells per well in 6-well cell culture plates. After 24 h the cells were treated with the desired reagents for durations as indicated. The cells were trypsinized and counted at 24 h, 48 h and 72 h after treatments using the automated cell counting system Luna (logos biosystems, Korea). IC50 for the cell population growth assay was the drug concentration inhibiting growth over the cell number at start of treatment by 50%, and was estimated by a three-parameter logistic model fit using the SAS NLMIXED procedure^72,73^.

### Clonogenic survival assay

For the measurement of clonogenic survival, A549 and H460 cells were seeded at a density of 4 × 10^5^ cells per well in 6-well cell culture plates for 24 h. To determine the effect of cisplatin or metformin on clonogenic survival, cells were treated with cisplatin (0–5 µM) or metformin (0–2 mM) for indicated times. Cells were plated in 6-well plates at a density of 100–1000 cells per well and colonies were counted after 10–14 days. To investigate the radiosensitizing effects of cisplatin, cells were irradiated with single doses of 0–6 Gy after 4 h of cisplatin (1 μM) treatment. Cells were plated in 6-well plates at a density of 100–1000 cells per well and colonies were counted after 10–14 days. To test whether metformin effects the radiosensitizing effect of cisplatin, cells were treated with metformin for 48 h followed by cisplatin for another 4 h. Cells were plated in 6-well plates at a density of 100–1000 cells per well and irradiated with single doses of 0–6 Gy. In the cisplatin, metformin and Compound-C combination experiments, cells were stimulated with Compound-C and metformin for 46 h and 48 h, respectively, followed by cisplatin (1 µM) treatment for 4 h. Cells were plated at a density of 100–10000 cells per well and irradiated with single doses of 0–6 Gy. Colonies were allowed to be formed at 37 °C in 5% CO_2_ for 10–14 days. Cells were fixed and stained (with 96% ethanol, 15% (w/v) Giemsa) and destained with distilled water. Colonies were counted that had >50 cells. The surviving fractions after the respective radiation dose are presented as a fraction of the growth of untreated colonies.

### Apoptotic cell death

Apoptotic fraction has been measured by caspase-3 activity by flow cytometry. A549 and H460 cells were treated for 24 h with metformin (1 mM for A549 and 2 mM for H460), subsequently cisplatin (1 µM) was added and cells were irradiated with 4 Gy and 20 Gy at 4 h thereafter. Cells were fixed and permeabilized in one step by adding 100 µl cytofix/cytoperm solution (BD Bioscience, Franklin Lakes, NJ) at 48 h after irradiation. Cells were washed in cytoperm/cytowash buffer (BD Bioscience) and incubated with specific rabbit anti-active caspase-3 antibody (BD Bioscience). Secondary antibody staining was performed using Alexa 488 coupled antibody. The fraction of positive cells was measured by flow cytometry.

### Immunoblotting

Cells were harvested and lysed in RIPA buffer (Thermo, Rockford, USA) supplemented with a 1x protease inhibitor cocktail (Roche, Mannheim, Germany) for 15 minutes at 4 °C. Equal amount of protein lysates were mixed with 4xLDS sample buffer (Invitrogen, Carlsbad, USA) and heated to 95 °C for 5 min prior to gel loading. After separation by NuPAGE 4–12% bis-tris or 7% tris-acetate gels (Invitrogen), proteins were transferred to PVDF blotting membranes (Invitrogen, CA, USA). Membranes were blocked in 5% defatted powdered milk/Tris–buffered saline 0.5% Tween 20 at room temperature for 1 h and incubated with the primary antibody over night at 4 °C. After washing, the membranes were incubated with the secondary antibody conjugated with horseradish peroxidase. Protein visualization was performed using SuperSignal West Femto Stable Peroxide Buffer and Luminol/Enhancer Solution (Thermo). Signal detection took place using ChemiDOC MP Imaging System (Biorad). The following antibodies were used: mouse mAb anti-ERCC1 1:1000 (Sc17809, Santa Cruz Biotechnology), rabbit mAb anti-pAMPK 1:1000 (2531, Cell Signalling), anti-rabbit IgG HRP-linked 1: 5000 (7074P2, Cell Signalling), rabbit pAb to GAPDH 1: 2000 (ab9485, abcam) or mouse mAb to GAPDH 1: 2000 (ab8245, abcam).

### Immunofluorescence microscopy

Cells were grown onto the 4- or 8-wells chamber slides. After treatment, cells were fixed with 4.5% formaldehyde for 20 min, permeabilized for 15 min in permeabilising buffer (100 mM Tris-HCl, pH 7.4; 50 mM EDTA, 0.5% Triton X-100) and thereafter blocked for 1 h at room temperature in the blocking solution (3% BSA, 0.1% Tween 20, 4xSSC). Cells were then incubated with the primary antibodies, i.e. rabbit anti-Rad51 1:500 (PC130-100, Calbiochem), rabbit anti-53BP1 1:500 (ab21083, abcam) and mouse anti γ-H2AX 1:500 (ab22551, abcam) overnight at 4 °C. The secondary antibodies goat anti-rabbit Cy3-conjugated antibody 1:500 (800-367-52, Jackson Immuno Research) and Alexa Fluor 488 goat anti-mouse 1:500 (A11017, life technologies) were applied together with 4′-,6-diamidino-2-phenylindole DAPI nuclear counterstain 1 µg/ml for 1 h at room temperature. Images were obtained using a fluorescence microscope Imager Z1 (Zeiss; Jena, Germany).

### Measurement of cisplatin-DNA adducts

Cisplatin-DNA adducts in NSCLC cells were measured as previously described _ENREF_79^70^, with minor modifications. Briefly, cells were directly cultured and grown onto the Superfrost Gold Slides (ThermoFisher). Cells were treated with increasing concentrations of cisplatin for 4 h, washed with PBS and cultured for further 20 h in drug free medium. After treatment, cells were fixed for 30 min in ice-cold methanol and subjected to proteolytic digestion with 60 µg/mL pepsin and 40 µg/mL proteinase K. Cells were blocked with non-specific binding sites with 5% (w/v) non-fat powdered milk in PBS at 37 °C and incubated at 4 °C overnight with a rat primary antibody (1:500) that specifically recognizes cisplatin-GpG DNA adducts (RC-18). The secondary antibodies anti-rat Cy3®-labelled antibody 1:500 (Dianova, Hamburg) were applied together with 4′-,6-diamidino-2-phenylindole DAPI nuclear counterstain 1 µg/ml (w/v) for 1 h at room temperature. Images were acquired on an Axioplan fluorescence microscope (Carl Zeiss GmbH, Göttingen, Germany). For the quantification of cisplatin-GpG (CP-GpG) DNA adducts, fluorescence signals were measured by quantitative digital image analysis using the ACAS 6.0 CytometryAnalysis System (ACAS II, Ahrens Electronics, Bargterheide, Germany). Levels of adducts in each sample were calculated as arbitrary fluorescence units (AFU’s), upon normalization of the integrated antibody-derived fluorescence from 250 individual nuclei/sample to the corresponding DNA content. Data are presented as the mean AFU ± 95% confidence interval (CI) from three to four independent experiments.

### Statistical analysis

All data were expressed as the mean ± standard error of the mean (S.E.M.). The statistical analysis between the groups was performed by analysis of variance (ANOVA), and the means of two data sets were compared by a two-tailed Students t**-**test using the GraphPrism 6 software (GraphPad, La Jolla, CA, USA). Colony data were analyzed using a general linear model with radiation dose (2Gy, 4Gy, 6Gy), and exposure to each drug, as well as interaction effects between the drugs, and the individual repeated experiment as independent classification variables (Procedure GLM, SAS/STAT 14.1, SAS Institute Inc. Version 9.4, Cary, NC, USA). Higher order effects were considered if significant. For each drug exposure combination, the ratio of counted colonies versus seeded cells at each radiation dose group was normalized by the plating efficiency of unirradiated control cells at the same drug exposure condition. The dependent observations were the logarithms of these normalized ratios from each experiment with at least three cell culture plates. The significance of an independent variable was assessed by ANOVA F-test. Statistical significance was set at a level of alpha = 0.05. Cisplatin-GpG DNA adduct levels were analyzed with a linear quadratic covariance model, allowing a linear and quadratic effect of cisplatin, a cisplatin dose independent metformin effect at cisplatin concentrations between 5 and 15 μM and an interaction effect analysis of metformin modifying the adduct dependence on the square of the cisplatin concentration (Procedure GLM, SAS/STAT 14.3, SAS Institute Inc.).

## Supplementary information

Figure S1
