# Supplementary material for: Metformin enhances the radiosensitizing effect of cisplatin in non-small cell lung cancer cell lines with different cisplatin sensitivities
Source: Sci Rep. 2019 Feb 4;9:1282. doi: 10.1038/s41598-018-38004-5 (PMC6361966; doi:10.1038/s41598-018-38004-5)
Supplement: Supplementary file 1 — Figure S1 [file 41598_2018_38004_MOESM1_ESM.docx]

**­­**

**Metformin enhances the radiosensitizing effect of cisplatin in non-small cell lung cancer cell lineswith different cisplatin sensitivities**

Muhammad Assad Riaz*^1^, Ali Sak^1^, Yasin Bahadir Erol^1^, Michael Groneberg^1^, Jürgen Thomale^2^, Martin Stuschke ^1^

^1^Department of Radiotherapy, University Hospital Essen, Essen, Germany, ^2^Institut of Cell Biology University Hospital Essen, Essen, Germany

**Supplementary material**

**Figure S1 B**


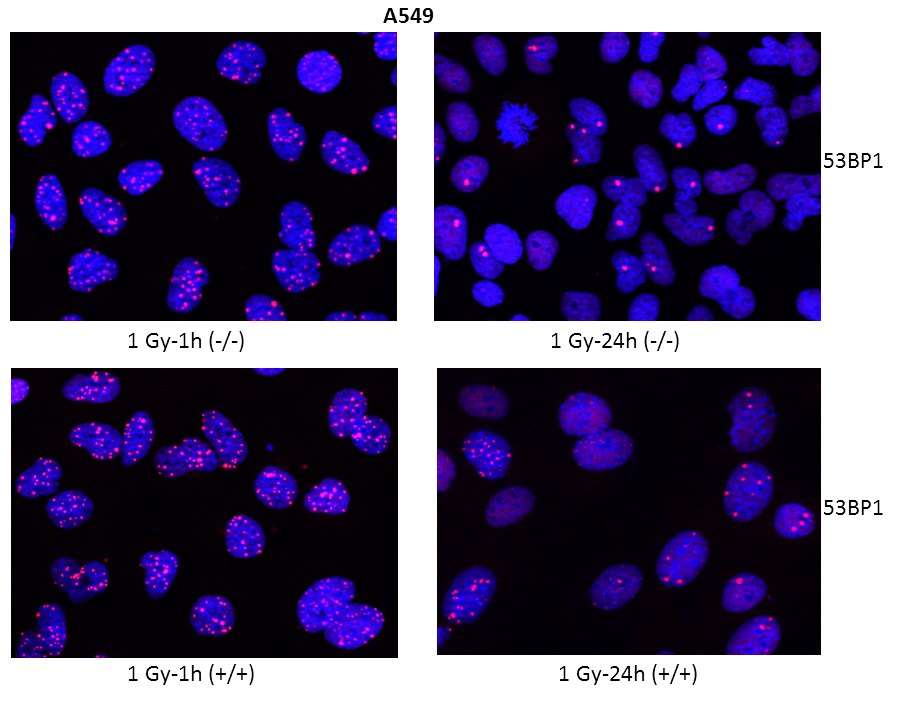


**Figure S1 C**


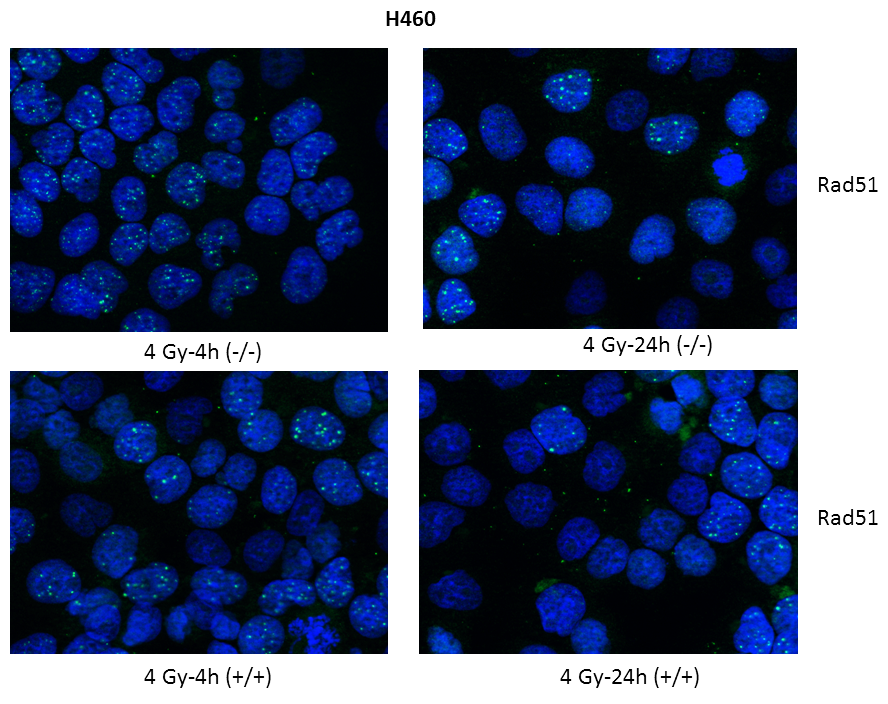


**Figure S1 D**


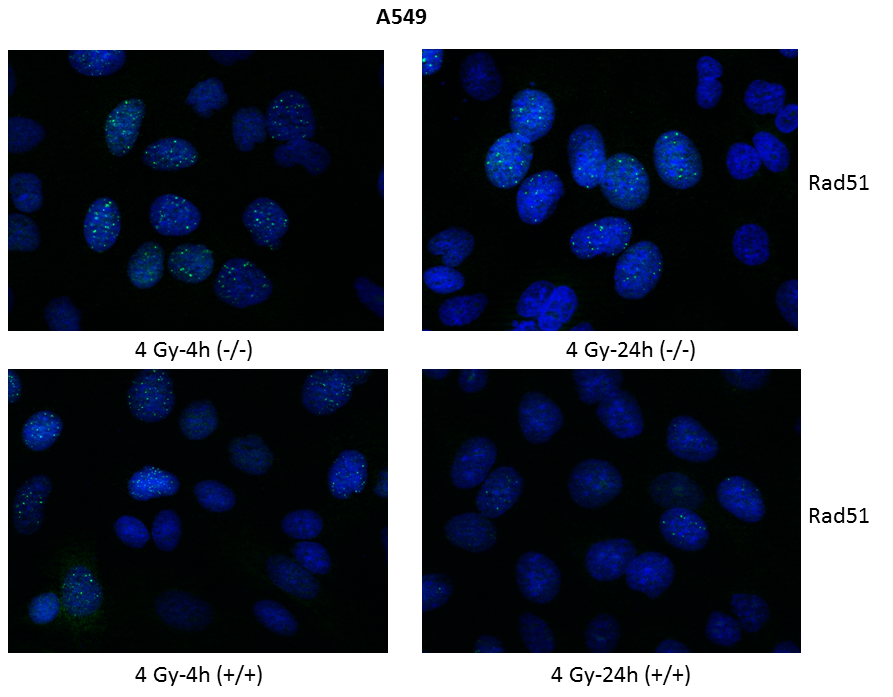


**Figure S1: Effect of cisplatin and metformin on radiation-induced foci formation in NSCLC cells**. Radiation**-**induced 53BP1 foci (A, B) and Rad51 foci (C, D) were measured in non-treated (-/-) and after combined treatment with metformin and cisplatin (+/+) in H460 (A, C) and A549 (B, D) cell lines. H460 and A549 cells were treated with 2 mM and 1 mM metformin for 24 h, respectively, followed by cisplatin (1µM) for another 4h as indicated. After irradiation (1 Gy), cells were fixed and stained for 53BP1 (1 h, 24 h) and Rad51 (4 h, 24 h). Images were acquired as a z-stack with a total of 20 focal plans and a step size of 0.27µm between planes. The maximum intensity images of each stack for each dose (ApoTome Microscope; Zeiss, Göttingen, Germany) with 640x magnification are shown. Blue (DAPI), red (53BP1 foci) and green (Rad51 foci).
